# Supplementary material for: A century of ecosystem change: human and seabird impacts on plant species extirpation and invasion on islands
Source: PeerJ. 2016 Jul 21;4:e2208. doi: 10.7717/peerj.2208 (PMC4963222; doi:10.7717/peerj.2208)
Supplement: Supplemental Information 1 — Includes a column for origin of species (native to coast British Columbia or exotic) and the current status on the island in comparison to 1896 (extinct, extant or introduced over the period between 1896 and 2012). [file peerj-04-2208-s001.docx]

Table S1: Species list of Mandarte Island, including a column for origin of species (native to coast British Columbia or exotic) and the current status on the island in comparison to 1896 (extinct, extant or introduced over the period between 1896 and 2012).

| **Scientific name** | **Origin** | **Current status in comparison to 1896** |
| --- | --- | --- |
| *Abies grandis* | native | extirpated |
| *Achillea millefolium* | native | extant |
| *Agrostis exarata* | native | extant |
| *Agrostis microphylla* | native | extirpated |
| *Aira caryophyllea* | exotic | introduced |
| *Aira praecox* | exotic | introduced |
| *Alnus viridis* | native | introduced |
| *Amelanchier alnifolia* | native | extant |
| *Amsinckia spectabilis* | native | extant |
| *Arabis eschscholtziana* | native | extirpated |
| *Arbutus menziesii* | native | extirpated |
| *Armeria maritima* | native | extirpated |
| *Barbarea verna* | exotic | extirpated |
| *Barbarea vulgaris* | exotic | extirpated |
| *Bromus pacificus* | native | extant |
| *Bromus rigidus* | exotic | introduced |
| *Bromus sitchensis* | native | extant |
| *Calandrinia ciniata* | native | extant |
| *Camassia leichtlinii* | native | extant |
| *Camassia quamash* | native | extirpated |
| *Capsella bursa-pastoris* | exotic | extirpated |
| *Cardamine nuttalli* | native | extirpated |
| *Cardamine oligosperma* | native | extirpated |
| *Castilleja miniata* | native | extirpated |
| *Cerastium arvense* | native | extant |
| *Chenopodium album* | exotic | introduced |
| *Cichorium intybus* | exotic | introduced |
| *Claytonia perfoliata* | native | extant |
| *Claytonia sibirica* | native | extirpated |
| *Crataegus monogyna* | exotic | extirpated |
| *Collinsia parviflora* | native | extant |
| *Dactylis glomerata* | exotic | introduced |
| *Daucus pusillus* | native | extirpated |
| *Elymus glaucus / hirsutus* | native | extant |
| *Elymus mollis* | native | extant |
| *Epilobium angustifolium* | native | extant |
| *Erythronium oregonum* | native | extant |
| *Festuca rubra* | native | extant |
| *Festuca saximontana/ovina* | native | extant |
| *Fritillaria affinis* | native | extant |
| *Galium aparine* | exotic | introduced |
| *Geranium molle* | exotic | introduced |
| *Glechoma hederacea* | exotic | introduced |
| *Grindelia integrifolia* | native | extant |
| *Hedera helix* | exotic | introduced |
| *Heuchera micrantha* | native | extant |
| *Holcus lanatus* | exotic | extant |
| *Holodiscus discolor* | native | extant |
| *Hordeum brachyantherum* | native | extant |
| *Hordeum jubatum* | exotic | introduced |
| *Hordeum murinum* | exotic | introduced |
| *Koeleria macrantha* | native | extirpated |
| *Lamium purpureum* | exotic | introduced |
| *Lathyrus japonicus var. maritimus* | native | extant |
| *Lathyrus nevadensis* | native | extant |
| *Lepidium virginicum* | native | extirpated |
| *Mahonia aquifolium* | native | extant |
| *Malus fusca* | native | extant |
| *Mimulus guttatus* | native | extant |
| *Mycelis muralis* | exotic | introduced |
| *Parentucellia viscosa* | exotic | introduced |
| *Plagiobothrys scouleri* | native | extant |
| *Poa annua* | exotic | introduced |
| *Poa pratensis* | exotic | introduced |
| *Polygonum newberryi* | native | extant |
| *Polygonum paronychia* | native | extirpated |
| *Polypodium glycyrrhiza* | native | extant |
| *Polystichum munitum* | native | extant |
| *Prunus emarginata* | native | extant |
| *Prunus virginiana* | native | extant |
| *Pseudotsuga menziesii* | native | extirpated |
| *Puccinellia nuttalliana* | native | extirpated |
| *Quercus garryana* | native | extant |
| *Ranunculus sceleratus* | native | extirpated |
| *Ribes divaricatum* | native | extant |
| *Rosa nutkana* | native | extant |
| *Rubus armeniacus* | exotic | introduced |
| *Rubus laciniatus* | exotic | introduced |
| *Rubus ursinus* | native | extant |
| *Rumex acetosella* | exotic | introduced |
| *Rumex crispus* | exotic | introduced |
| *Sagina maxima* | native | extant |
| *Salix lucida* | native | extant |
| *Salix scouleriana* | native | extant |
| *Sambucus racemosa* | native | introduced |
| *Sanicula crassicaulis* | native | extant |
| *Sedum spathulifolium* | native | extant |
| *Senecio sylvaticus* | exotic | introduced |
| *Sisymbrium officinale* | exotic | introduced |
| *Solanum dulcamara* | exotic | introduced |
| *Sonchus asper* | exotic | introduced |
| *Sorbus aucuparia* | exotic | introduced |
| *Stellaria calycantha* | native | extirpated |
| *Stellaria media* | exotic | introduced |
| *Symphoricarpos albus* | native | extant |
| *Tellima grandiflora* | native | extant |
| *Urtica dioica* | exotic | introduced |
| *Urtica urens* | exotic | introduced |
| *Vicia americana* | native | extant |
| *Vulpia bromoides* | exotic | introduced |
