# Supplementary material for: A century of ecosystem change: human and seabird impacts on plant species extirpation and invasion on islands
Source: PeerJ. 2016 Jul 21;4:e2208. doi: 10.7717/peerj.2208 (PMC4963222; doi:10.7717/peerj.2208)
Supplement: Supplemental Information 2 — Vegetation surveys have been conducted in 2007 and 2008 (Bennett et al., 2012) and 2012 (this study). Islands are classified as either supporting seabird colonies (‘gull islands’) or not (‘non-gull islands’). [file peerj-04-2208-s002.docx]

Table S2: List of islands on which vegetation surveys have been conducted in 2007 and 2008 (Bennett et al. 2012) and 2012 (this study). Islands are classified as either supporting seabird colonies (‘gull islands’) or not (‘non-gull islands’).

| **Island** | **type** | **no. of plots** | **Size (ha)** | **data collection** |
| --- | --- | --- | --- | --- |
| Arbutus Island | gull island | 4 | 0.29 | 2012 |
| Mandarte Island | gull island | 14 | 6.80 | 2012 |
| unnamed islet south of Mandarte | gull island | 3 | 0.18 | 2012 |
| Alec Rocks | non-gull island | 4 | 0.40 | 2007-2008 |
| Amelia Island | non-gull island | 3 | 4.02 | 2007-2008 |
| Big Isabella | non-gull island | 2 | 0.34 | 2007-2008 |
| Boulder Island | non-gull island | 2 | 1.81 | 2007-2008 |
| Channel Middle | non-gull island | 2 | 0.59 | 2007-2008 |
| Coon Island | non-gull island | 2 | 1.13 | 2007-2008 |
| Dock North | non-gull island | 2 | 0.63 | 2007-2008 |
| Dock South | non-gull island | 3 | 0.62 | 2007-2008 |
| Fortress Island | non-gull island | 2 | 0.70 | 2007-2008 |
| Little Channel | non-gull island | 1 | 0.34 | 2007-2008 |
| Long Islet | non-gull island | 2 | 0.40 | 2007-2008 |
| McConnell Island | non-gull island | 5 | 11.60 | 2007-2008 |
| North Ada | non-gull island | 3 | 3.66 | 2007-2008 |
| Reay Island | non-gull island | 3 | 0.65 | 2007-2008 |
| Rum Island | non-gull island | 2 | 0.33 | 2007-2008 |
| Sentinel Island | non-gull island | 3 | 6.61 | 2007-2008 |
| Skull Islet | non-gull island | 3 | 1.14 | 2007-2008 |
| Small Isabella | non-gull island | 1 | 0.26 | 2007-2008 |
| South Ada | non-gull island | 3 | 3.83 | 2007-2008 |
| South Winchelsea | non-gull island | 2 | 11.53 | 2007-2008 |
| Wallace Point Island | non-gull island | 3 | 3.01 | 2007-2008 |
| Yeo Island | non-gull island | 4 | 1.93 | 2007-2008 |
